# Supplementary material for: Identification and structural characterization of small molecule inhibitors of PINK1
Source: Sci Rep. 2024 Apr 2;14:7739. doi: 10.1038/s41598-024-58285-3 (PMC10987619; doi:10.1038/s41598-024-58285-3)
Supplement: Supplementary file 1 — Supplementary Figures. [file 41598_2024_58285_MOESM1_ESM.pdf]

Supporting information:

Identification and structural characterization of small molecule inhibitors of PINK1

Shafqat Rasool[1*], Tara Shomali[1*], Luc Truong[1], Nathalie Croteau[1], Simon Veyron[1], Bernardo A. Bustillos[2], Wolfdieter Springer[2,3], Fabienne C. Fiesel[2,3], Jean-François Trempe[1#]

[1] Department of Pharmacology & Therapeutics, Centre de Recherche en Biologie Structurale, and Structural Genomics Consortium, McGill University, 3655 Prom Sir William Osler, H3G 1Y6, Montréal, Québec, Canada

[2] Department of Neuroscience, Mayo Clinic, Jacksonville, FL 32224, USA

[3] Neuroscience PhD Program, Mayo Clinic Graduate School of Biomedical Sciences, Jacksonville, FL 32224, USA

* These authors contributed equally to this work

# Correspondence: jeanfrancois.trempe@mcgill.ca

Running title: Structural characterization of PINK1 inhibitors

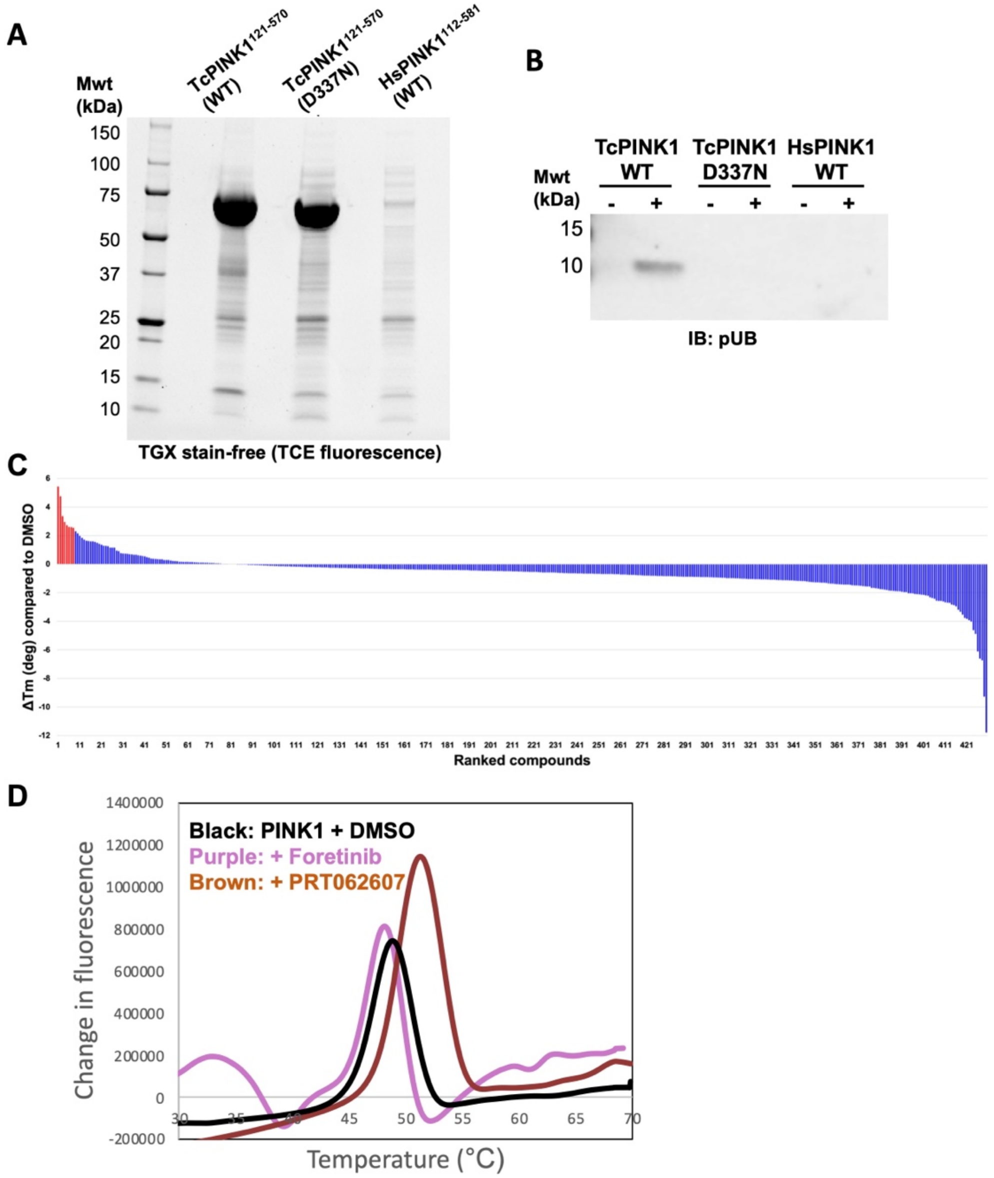

Supplemental Figure S1. Thermal shift assay screening of TcPINK1 targeting molecules. (A) Affinity purification of GST-PINK1 constructs expressed from same amount of E. coli culture. All constructs were eluted with equal volumes of elution buffer and equal volume was loaded on SDS-PAGE. D337N is a kinase-dead variant of TcPINK1. **(B)** Kinase assays were performed with 2 µM of GST-PINK1 constructs and 20 µM of ubiquitin. Products were resolved by SDS-PAGE and immunoblotted for phospho-UbS65. Results show that TcPINK1 WT phosphorylates ubiquitin, whereas TcPINK1-D337N and HsPINK1 do not. **(C)** Thermal shift (*ΔTm*) values obtained for TcPINK1 incubated with Sypro Orange and 100 µM compound derived from a subset of a SelleckChem kinase small molecule library (430 compounds). Compounds were ranked by *ΔTm* value. Top ranked compounds are shown in red. **(D)** Example of thermal denaturation data obtained for TcPINK1 incubated with DMSO (baseline), Foretinib (negative control) and PRT062607 (hit, positive *ΔTm*). The first derivative of the change in fluorescence is plotted as a function of the temperature. The Tm corresponds to the peak of the change in fluorescence (inflection point).

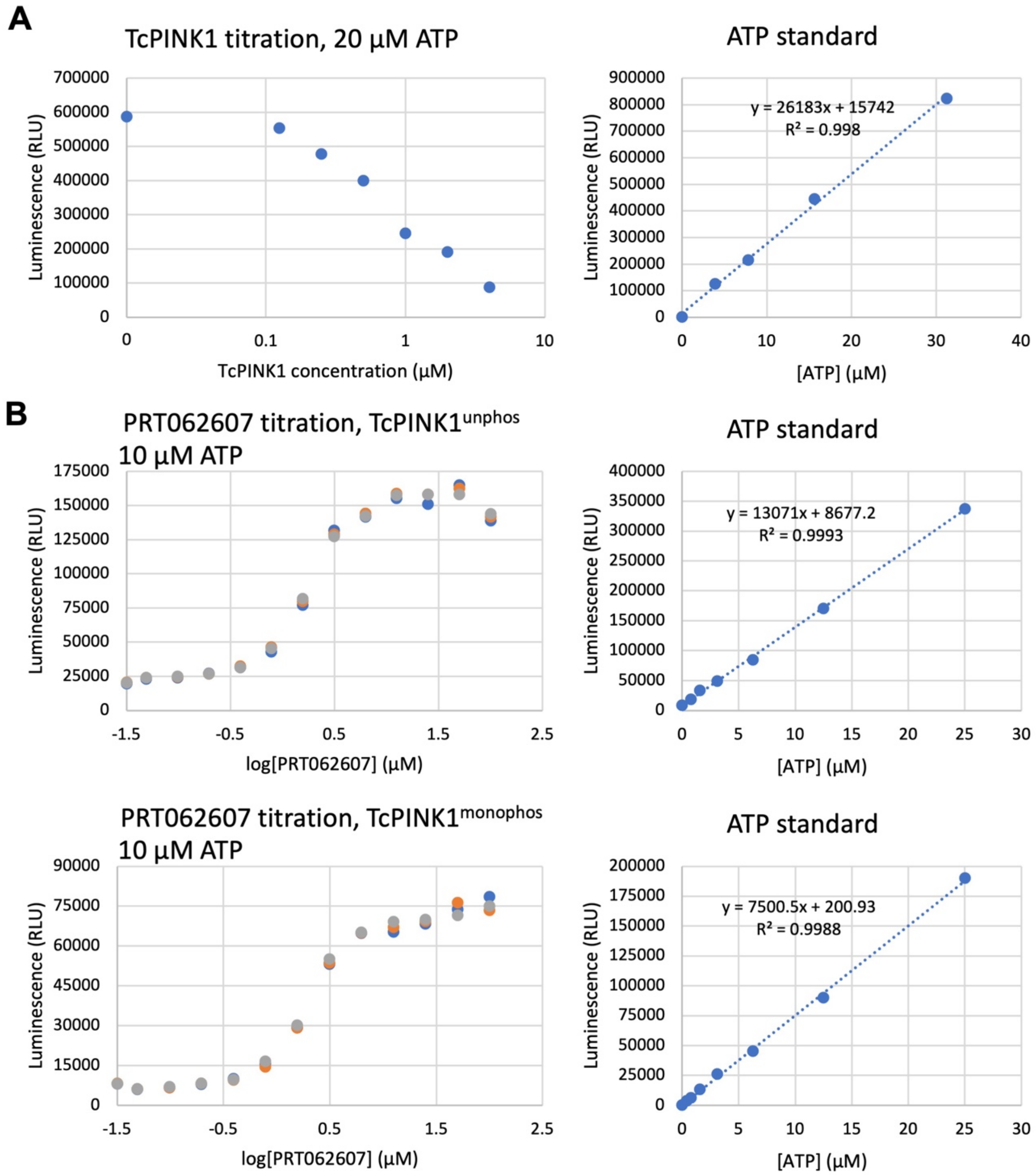

Supplemental Figure S2. Optimization and analysis of the Kinase Glo assay for IC_{50} determination. (A) Luminescence values observed after incubating 20 µM ATP for 5 min in the presence of difference TcPINK1 concentrations (left). The midpoint EC_{50} is around 1 µM TcPINK1. The ATP standard curve used in this assay is shown to the right and shows linearity for the entire range of luminescence observed. **(B)** Raw luminescence data used for IC_{50} determination (see Figure 2D). Different concentrations of PRT062607 was incubated with 1 µM TcPINK1$^{121-570}$ (unphosphorylated or mono-phosphorylated) and 10 µM ATP for 5 min. Reactions were performed in triplicates. The ATP standard curve for each experiment is shown on the right.

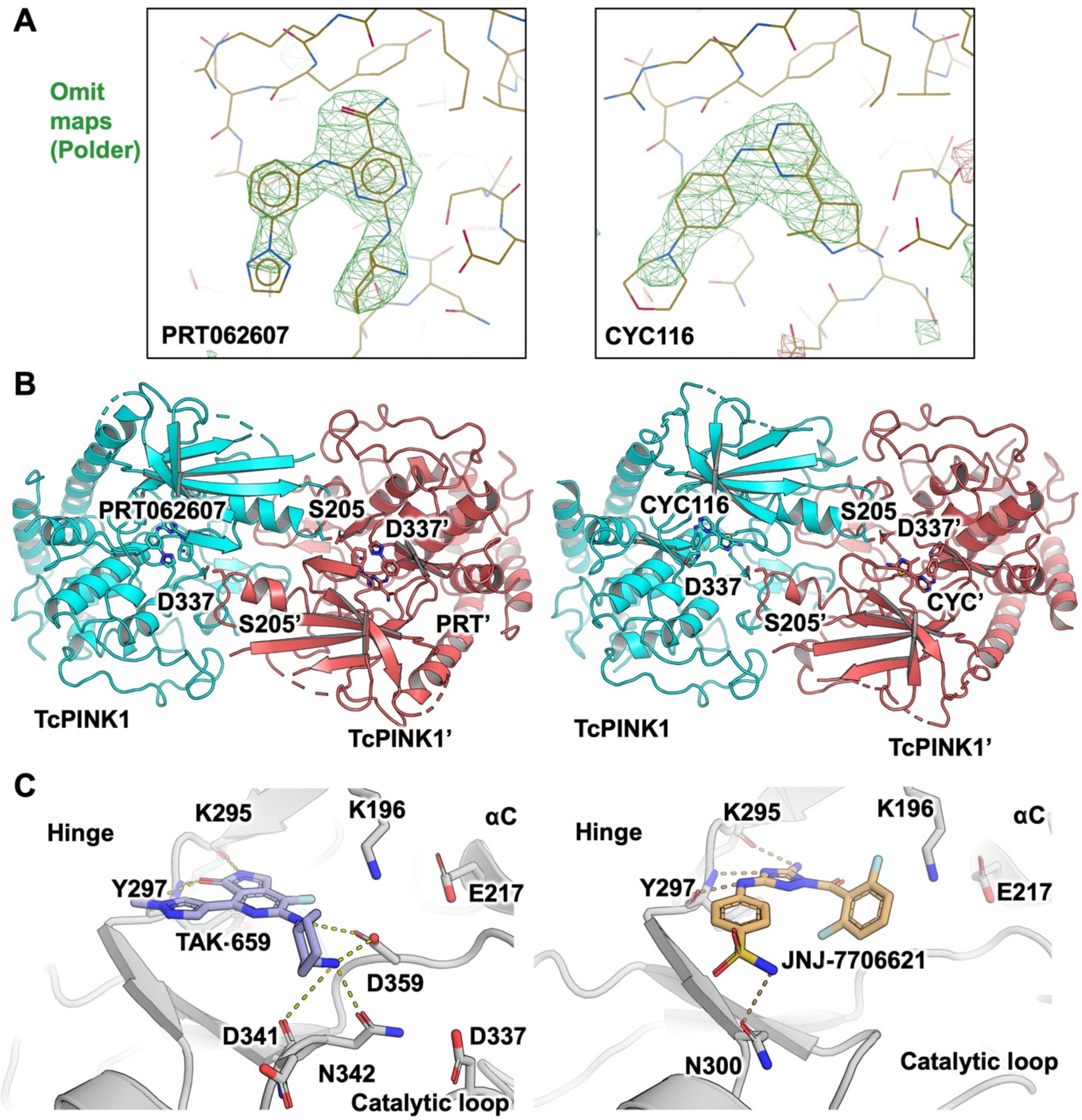

Supplemental Figure S3. Structural analysis of TcPINK1 binding to different inhibitors. (A) Polder ligand omit maps (3.5 σ) calculated from the crystal structures TcPINK$^{121\text{-}570}$ bound to PRT062607 (left) and CYC116 (right). **(B)** Crystallographic symmetry reveals face-to-face trans autophosphorylation complex in both PRT060207 and CYC116-bound TcPINK1, as observed previously for the apo and AMP-PN bound structures (PDB: 7MP8 and 7MP9). **(C)** Docking models of TcPINK1 bound to TAK-659 (left) or JNJ-7706621 (right), showing their interactions with the hinge, A-loop and catalytic loop. The apo structure of TcPINK1$^{121\text{-}570}$ (PDB 7MP8) was used for docking the small molecules using the software *DiffDock*.

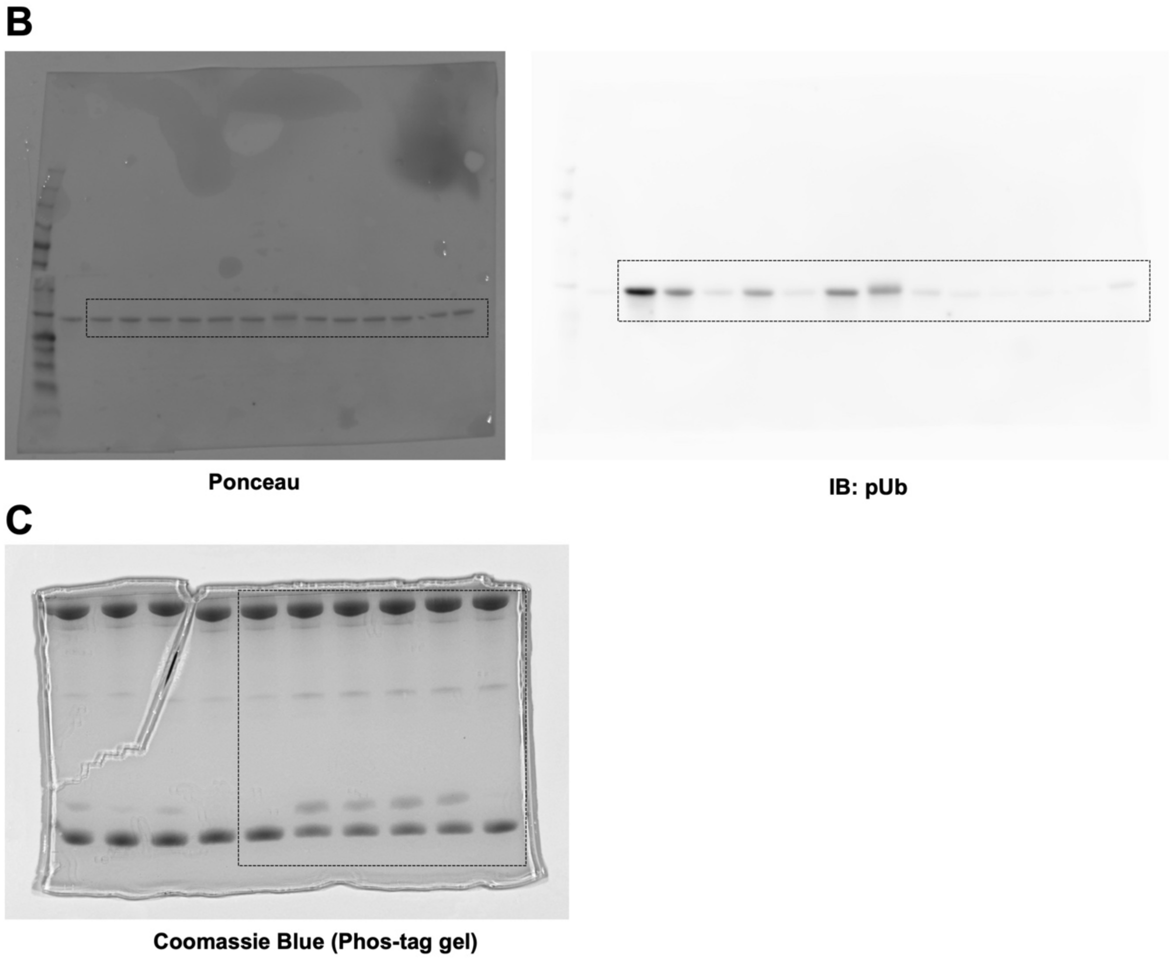

Supplemental Figure S4. Uncropped images for the immunoblots shown in Figure 1B,C. All lanes are loaded in the same order as the corresponding figure in the paper.

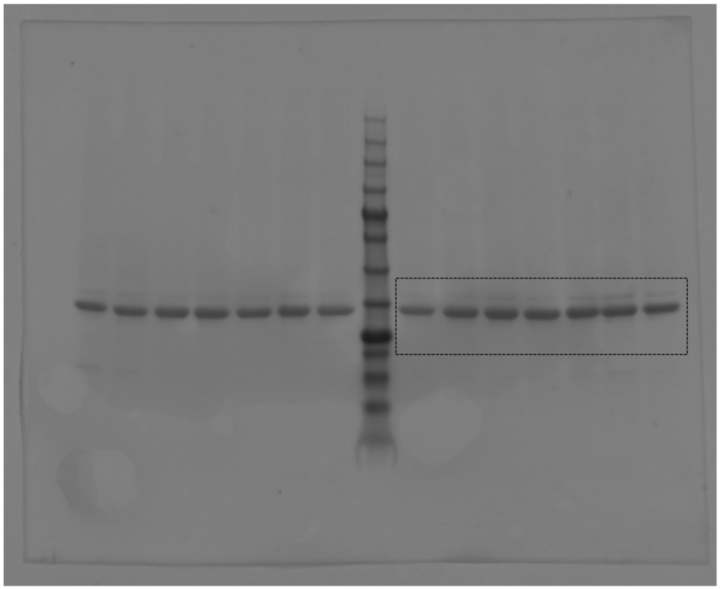

Ponceau

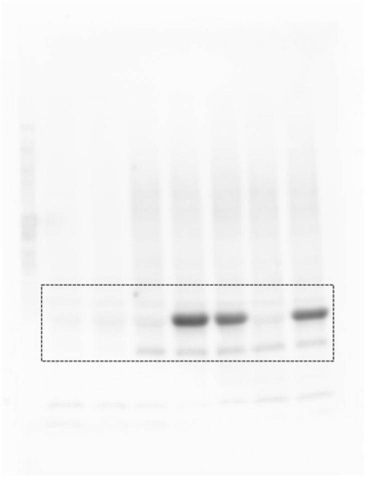

IB: pUb

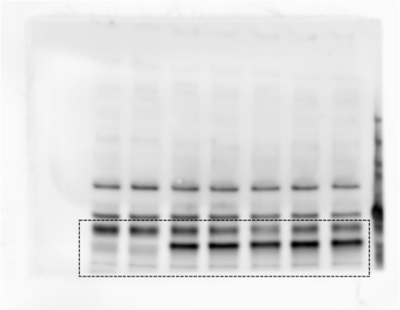

IB: PINK1

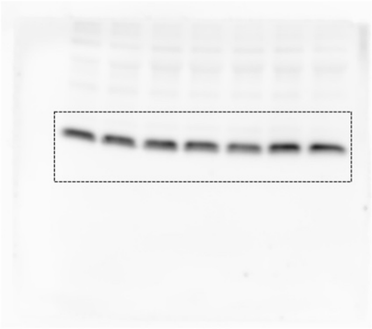

IB: CytC

Supplemental Figure S5. Uncropped images for the immunoblots shown in Figure 3A. All lanes are presented in the same order as the corresponding figure in the paper.

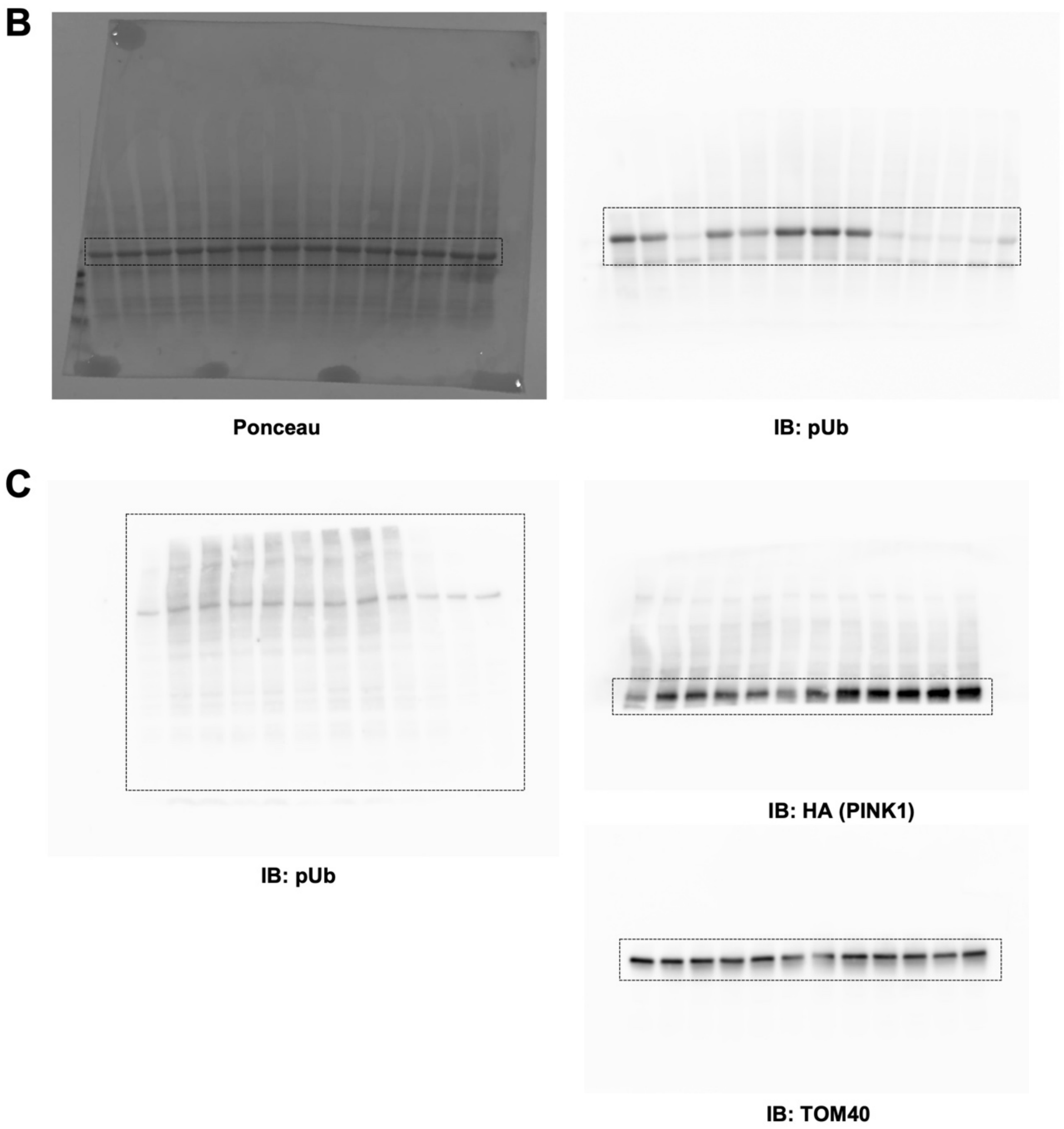

Supplemental Figure S6. Uncropped images for the immunoblots shown in Figure 3B,C. All lanes are presented in the same order as the corresponding figure in the paper.

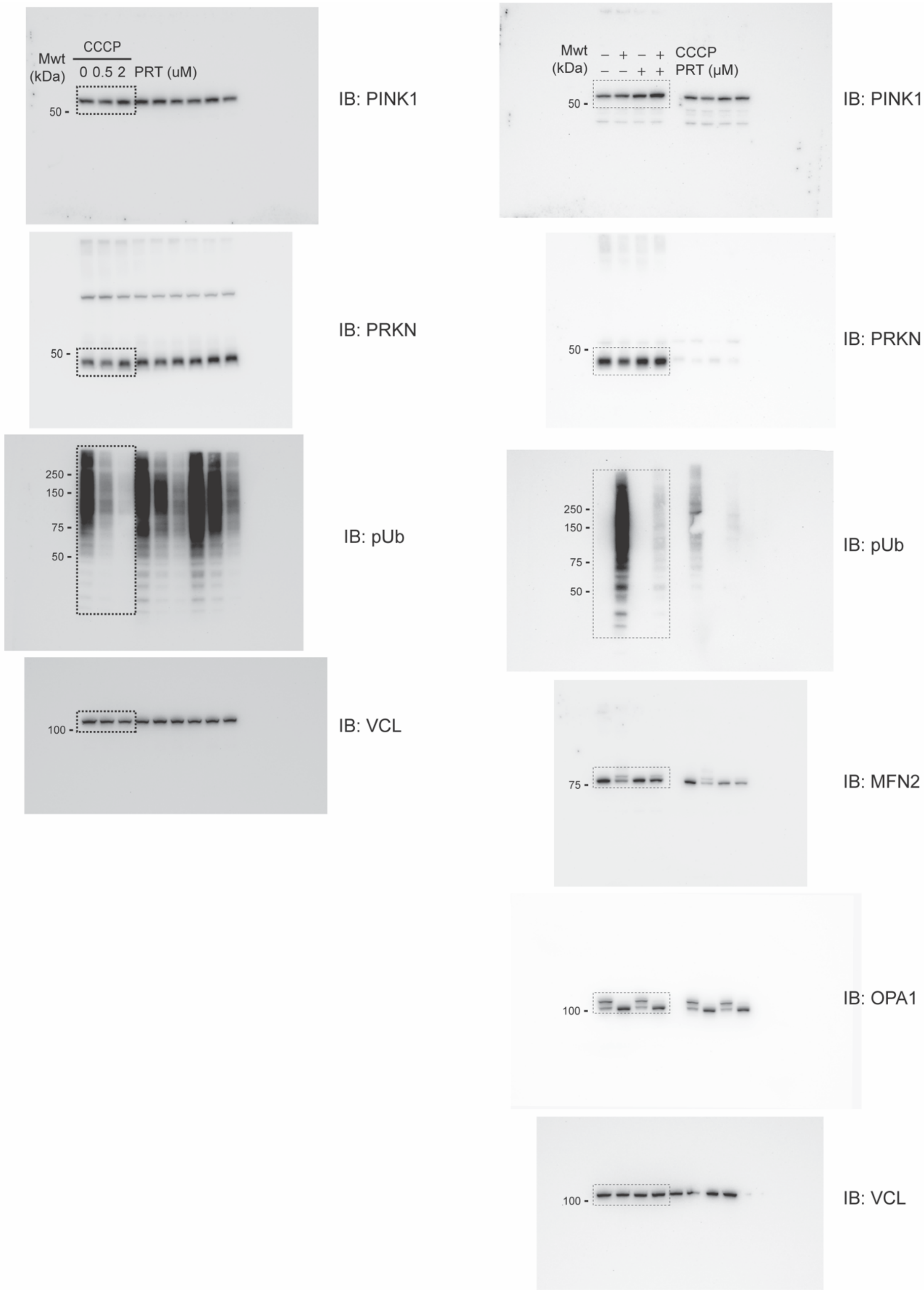

Supplemental Figure S7. Uncropped images for the immunoblots shown in Figure 3D.
